# Supplementary material for: Identifying regulators of aberrant stem cell and differentiation activity in colorectal cancer using a dual endogenous reporter system
Source: Nat Commun. 2024 Mar 12;15:2230. doi: 10.1038/s41467-024-46285-w (PMC10933491; doi:10.1038/s41467-024-46285-w)
Supplement: Supplementary file 3 — Description of Additional Supplementary Files [file 41467_2024_46285_MOESM3_ESM.pdf]

Description of Additional Supplementary Files

Supplementary Datasets

Supplementary Data 1. Sequences of sgRNA and homology arm for SOX9 and KRT20 knock-in

Supplementary Data 2. List of 78 epigenetic regulators from 5 families and their associated drugs

Supplementary Data 3. List of 76 sgRNAs and 154 shRNAs for control libraries and 542 sgRNAs for the epigenetic library

Supplementary Data 4. List of sequences used in the validation

Supplementary Data 5. List of antibodies used for the western blot
