# Supplementary material for: Identifying regulators of aberrant stem cell and differentiation activity in colorectal cancer using a dual endogenous reporter system
Source: Nat Commun. 2024 Mar 12;15:2230. doi: 10.1038/s41467-024-46285-w (PMC10933491; doi:10.1038/s41467-024-46285-w)
Supplement: Supplementary file 9 — Reporting Summary [file 41467_2024_46285_MOESM9_ESM.pdf]

nature portfolio

Corresponding author(s): Nilay Sethi

Last updated by author(s): Feb 13, 2024

Reporting Summary

Nature Portfolio wishes to improve the reproducibility of the work that we publish. This form provides structure for consistency and transparency in reporting. For further information on Nature Portfolio policies, see our Editorial Policies and the Editorial Policy Checklist.

Statistics

For all statistical analyses, confirm that the following items are present in the figure legend, table legend, main text, or Methods section.

n/a	Confirmed	
☐	☒	The exact sample size (*n*) for each experimental group/condition, given as a discrete number and unit of measurement
☐	☒	A statement on whether measurements were taken from distinct samples or whether the same sample was measured repeatedly
☐	☒	The statistical test(s) used AND whether they are one- or two-sided<br>*Only common tests should be described solely by name; describe more complex techniques in the Methods section.*
☒	☐	A description of all covariates tested
☐	☒	A description of any assumptions or corrections, such as tests of normality and adjustment for multiple comparisons
☐	☐	A full description of the statistical parameters including central tendency (e.g. means) or other basic estimates (e.g. regression coefficient) AND variation (e.g. standard deviation) or associated estimates of uncertainty (e.g. confidence intervals)
☐	☒	For null hypothesis testing, the test statistic (e.g. *F*, *t*, *r*) with confidence intervals, effect sizes, degrees of freedom and *P* value noted<br>*Give P values as exact values whenever suitable.*
☒	☐	For Bayesian analysis, information on the choice of priors and Markov chain Monte Carlo settings
☒	☐	For hierarchical and complex designs, identification of the appropriate level for tests and full reporting of outcomes
☐	☒	Estimates of effect sizes (e.g. Cohen's *d*, Pearson's *r*), indicating how they were calculated

Our web collection on statistics for biologists contains articles on many of the points above.

Software and code

Policy information about availability of computer code

Data collection

See methods "Flow cytometry and cell sorting (FACS)","RNA isolation and qPCR", "PERTURB-seq ","Immunoblot analysis","CRC cell line xenograft in vivo tumor model". Specific software includes Nikon NIS elements software, FlowJo_v10.8.0, FIJI.

Data analysis

Experiments were performed in triplicate. Data are represented as mean±s.d unless indicated otherwise. For each experiment, either independent biological or technical replicates were conducted as noted in the figure legends and were repeated with similar results. Statistical analysis was performed using Microsoft Office or Prism 8.0 (GraphPad) statistical tools. Pairwise comparisons between group means were performed using an unpaired two-tailed Student's t-test or Kruskal–Wallis test as appropriate unless otherwise indicated. Multi-group comparison of means was performed using one-way ANOVA with post-hoc Tukey's HSD test as appropriate unless otherwise indicated. For all experiments, the variance between comparison groups was found to be equivalent.

To process and analyze CRISPR screen data, the following softwares, packeges and modules were used: Python v.3.9.13 (Pandas v.1.5.3, Numpy v.1.26.1, Seaborn v.0.11.2, Scipy v.1.11.3, Matplotlib v.3.7.2, Statsmodels v.0.13.2, GSEApy v.1.1.1), MaGeCK v.0.5, STRING v.12.0.

FASTQ files of GEX and CRISPR libraries were processed with default parameters on 10X Genomics Cell Ranger pipeline (v7.0.0) using GRCh38-2020-A reference. Processed data were then analyzed with Seurat (v4.1.1) 77and its extension Mixscape 78 under "R" (version 4.2.2) environment.

Code associated with the CRISPR screens using endogenous reporter system are available at https://github.com/davidchen0420/Endogenous_Reporter

For manuscripts utilizing custom algorithms or software that are central to the research but not yet described in published literature, software must be made available to editors and reviewers. We strongly encourage code deposition in a community repository (e.g. GitHub). See the Nature Portfolio guidelines for submitting code & software for further information.

Data

Policy information about availability of data

All manuscripts must include a data availability statement. This statement should provide the following information, where applicable:

- Accession codes, unique identifiers, or web links for publicly available datasets
- A description of any restrictions on data availability
- For clinical datasets or third party data, please ensure that the statement adheres to our policy

Data sets generated in this study have been deposited in the Gene Expression Omnibus (GEO) database under accession code GSE236257. CRISPR screen read count data and analysis code can be found at GSE236257 and https://github.com/davidchen0420/Endogenous_Reporter. Sequencing reads are aligned to the human genome build hg38. All figures have associated raw data in source files.

Research involving human participants, their data, or biological material

Policy information about studies with human participants or human data. See also policy information about sex, gender (identity/presentation), and sexual orientation and race, ethnicity and racism.

Reporting on sex and gender	No selection bias was present.
Reporting on race, ethnicity, or other socially relevant groupings	No selection bias was present.
Population characteristics	Research specimen were blinded to clinical information related to the patient.
Recruitment	Patient with colorectal cancer who underwent surgery was recruited post-informed consent for sample collection for research purposes under IRB protocol 13-189 and 14-408. No selection bias was present.
Ethics oversight	The Internal Review Board of the Dana Farber Cancer Institute, Boston, Massachusetts, USA (protocol 13-189)

Note that full information on the approval of the study protocol must also be provided in the manuscript.

Field-specific reporting

Please select the one below that is the best fit for your research. If you are not sure, read the appropriate sections before making your selection.

☒ Life sciences ☐ Behavioural & social sciences ☐ Ecological, evolutionary & environmental sciences

For a reference copy of the document with all sections, see nature.com/documents/nr-reporting-summary-flat.pdf

Life sciences study design

All studies must disclose on these points even when the disclosure is negative.

Sample size	For the reporter screen, minimum of 6 guides targeting each gene were used and a minimum of 500 cells were captured for each guide in the library. The sample size is sufficient to avoid sampling bias (as recommended in the field, PMID: 28333914). For xenograft experiments in Figure 5, we used minimum 8 mice per group based on our and other prior work.
Data exclusions	Entire experiments were not included if control line did not behave as expected (e.g. poor quality after thawing).
Replication	All findings were reproducible. More than 1 biological replicate was used for all experiments. Sample sizes were large enough to sure appropriate representation of the population behavior.
Randomization	Randomization was performed at the beginning of xenograft experiments in Figure 5. Most mice received control and experimental cells in each flank, controlling for mouse to mouse variation. No other experiments that required randomization
Blinding	All histopathology scoring and assessments were performed in a blinded fashion. No other experiments required blinded evaluation.

Reporting for specific materials, systems and methods

We require information from authors about some types of materials, experimental systems and methods used in many studies. Here, indicate whether each material, system or method listed is relevant to your study. If you are not sure if a list item applies to your research, read the appropriate section before selecting a response.

Materials & experimental systems

n/a	Involved in the study
☐	☒ Antibodies
☐	☒ Eukaryotic cell lines
☒	☐ Palaeontology and archaeology
☐	☒ Animals and other organisms
☒	☐ Clinical data
☒	☐ Dual use research of concern
☒	☐ Plants

Methods

n/a	Involved in the study
☒	☐ ChIP-seq
☐	☒ Flow cytometry
☒	☐ MRI-based neuroimaging

Antibodies

Antibodies used: Supplementary Data 5 listing the antibodies used in this study. anti-SOX9 (1:600, CST, #82630), anti-KRT20 (1:500, CST, #13063), anti-CD26/DPP4 (1:200, #MA5-32643), anti-Lgr5 (1:200, # TA503316), anti-SMARCB1 (1:200, CST, #91735) and anti-GAPDH (1:3000, CST,# 2118)

Validation: Antibodies were validated by a combination of manufacturer, evaluating expected size on immunoblots, and genetic manipulation whenever possible. Liang et. al., An Enhancer-Driven Stem Cell-Like Program Mediated by SOX9 Blocks Intestinal Differentiation in Colorectal Cancer. Gastroenterology. 2022

Eukaryotic cell lines

Policy information about cell lines and Sex and Gender in Research

Cell line source(s): The human colorectal cancer cell lines (HT-115, HT-29 (HTB-38) and LS180(CL-187)) and HEK293T were obtained from CCLE core facility at the Broad Institute and used at early passage for the experiments. All mouse and human organoids were established in our laboratory from in vivo experiments outlined in the study.

Authentication: All cell lines underwent STR authentication testing.

Mycoplasma contamination: Mycoplasma testing was performed every 3 months and found to be negative on each check.

Commonly misidentified lines (See ICLAC register): N/A, our study did not involve commonly misidentified cell lines.

Animals and other research organisms

Policy information about studies involving animals; ARRIVE guidelines recommended for reporting animal research, and Sex and Gender in Research

Laboratory animals: All xenograft experiments were performed in Foxn1nu mice. Ambient temperature is a range between 68 to 75 degrees Fahrenheit. Humidity is between 30 and 70 percent. Most animal rooms follow a light: dark cycle of 12:12.

Wild animals: No wild animals were used in the study.

Reporting on sex: Equal sex ratios were used for in vivo experiments.

Field-collected samples: No field collected samples were used in the study.

Ethics oversight: All procedures involving mice and experimental protocols were approved by Institutional Animal Care and Use Committee (IACUC) of Dana-Farber Cancer Institute (11-009)

Note that full information on the approval of the study protocol must also be provided in the manuscript.

Plants

Seed stocks	*Report on the source of all seed stocks or other plant material used. If applicable, state the seed stock centre and catalogue number. If plant specimens were collected from the field, describe the collection location, date and sampling procedures.*
Novel plant genotypes	*Describe the methods by which all novel plant genotypes were produced. This includes those generated by transgenic approaches, gene editing, chemical/radiation-based mutagenesis and hybridization. For transgenic lines, describe the transformation method, the number of independent lines analyzed and the generation upon which experiments were performed. For gene-edited lines, describe the editor used, the endogenous sequence targeted for editing, the targeting guide RNA sequence (if applicable) and how the editor was applied.*
Authentication	*Describe any authentication procedures for each seed stock used or novel genotype generated. Describe any experiments used to assess the effect of a mutation and, where applicable, how potential secondary effects (e.g. second site T-DNA insertions, mosiacism, off-target gene editing) were examined.*

Flow Cytometry

Plots

Confirm that:

☒ The axis labels state the marker and fluorochrome used (e.g. CD4-FITC).

☒ The axis scales are clearly visible. Include numbers along axes only for bottom left plot of group (a 'group' is an analysis of identical markers).

☒ All plots are contour plots with outliers or pseudocolor plots.

☒ A numerical value for number of cells or percentage (with statistics) is provided.

Methodology

Sample preparation	Samples were prepared as described in methods section "Flow cytometry and cell sorting (FACS)".
Instrument	FACSAria III platform (BD) at DFCI Flow-core facility
Software	FloJo software
Cell population abundance	All gating parameters were fixed when analyzing control versus experimental groups.
Gating strategy	please see methods "Flow cytometry and cell sorting (FACS)" and Figure 1d.

☒ Tick this box to confirm that a figure exemplifying the gating strategy is provided in the Supplementary Information.
